# Heterogeneous Acupuncture Effects of Taixi (KI3) on Functional Connectivity in Healthy Youth and Elder: A Functional MRI Study Using Regional Homogeneity and Large-Scale Functional Connectivity Analysis

**DOI:** 10.1155/2020/8884318

**Published:** 2020-12-08

**Authors:** Linlu He, Guangxiang Chen, Ruwen Zheng, Yan Hu, Xiu Chen, Jianghai Ruan

**Affiliations:** ^1^Department of Geriatrics, The Affiliated Hospital of Southwest Medical University, Luzhou 646000, China; ^2^Department of Neurology, The Affiliated Hospital of Southwest Medical University, Luzhou 646000, China; ^3^Laboratory of Neurological Diseases and Brain Function, Luzhou 646000, China; ^4^Department of Radiology, The Affiliated Hospital of Southwest Medical University, Luzhou 646000, China; ^5^Department of Acupuncture and Moxibustion, Dongfang Hospital, Beijing University of Chinese Medicine, Beijing 100071, China

## Abstract

Heterogeneous neurological responses of acupuncture between different groups have been proposed by previous studies but rarely studied. The study described here was designed to explore the divergence of acupuncture at Taixi (KI3) on spontaneous activity of brain regions and functional connectivity (FC) between healthy youth and elder with functional magnetic resonance imaging (fMRI). 20 healthy young volunteers and 20 healthy elders underwent 10-minute-resting-state fMRI before acupuncture, and then acupuncture at Taixi (KI3) for 3 minutes; after withdrawing the needles, volunteers underwent a second fMRI scan for 10 minutes. Regional homogeneity (ReHo) and large-scale FC analysis using Power 264 atlas were utilized to analyze the changes of brain spontaneous activity. Compared with the resting state, the decreased ReHo after acupuncture at KI3 in both groups were concentrated in the left postcentral, right paracentral lobule, and right SMA. Moreover, the subjects in the HY group showed declined ReHo in brain regions involving the right lingual and precentral. However, those subjects in the HE group presented decreased ReHo in the right postcentral and precentral, left supramarginal gyrus and SMA, and both cingulum middle after needling in KI3. Compared with the resting state, the HY group in the postneedling state showed lower mean intranetwork FC in sensory/somatomotor and subcortical network. And the internetwork FC between sensory/somatomotor and dorsal attention had significantly decreased after acupuncture. Furthermore, the internetwork FC between subcortical and dorsal attention and between subcortical and cerebellar showed the most obvious elevations after needling in the HY group. In the elder group, both FCs of internetwork and intranetwork primarily involving sensory/somatomotor, cingulo-opercular, and dorsal attention were declined after acupuncture. These results indicated that acupuncture at KI3 had heterogeneous acupuncture effects in different age groups. Our study led to converging evidence supporting the acupuncture effect segregation of different condition subjects and supporting evidence for prevention and treatment with acupuncture in the future.

## 1. Introduction

Acupuncture has been applied in China for thousands of years and has emerged rapidly gaining popularity in Western alternative and complementary medicine practice for its therapeutic effects [1]. Many studies have demonstrated that acupuncture plays an important role in stroke rehabilitation, pain relief, cognitive function improvement, etc. [2–4]. The biological mechanism, however, remains to be clarified. The blood oxygenation level-dependent functional MRI (fMRI) techniques have shed light on the issues involving the mechanism of acupuncture, and the application of fMRI in the research of acupuncture has made abundant achievements in recent years.

Recently, cumulative evidence from fMRI studies has shown that stimulating acupoint could induce changes of brain functional connectivity [5]. For example, needling LV3 and LI4 points on patients with Alzheimer's Disease could enhance the functional connection in areas related to the hippocampus, which might be the potential mechanism of acupuncture to improve Alzheimer's Disease [6]. In stroke patients, acupuncture at TE5 could increase the cooperation of bilateral sensorimotor networks [7].

Taixi (KI3), one of the key acupoints of kidney meridian in the theory of Traditional Chinese Medicine (TCM), has been used to treat patients with cognitive impairment [8], which has been supported by acupuncture studies in healthy volunteers: needling acupoint KI3 could increase connectivity between cognition-related regions [9]. Moreover, a previous fMRI study [10] has found that acupuncture at the same acupoint of different people has a different impact on brain functional connectivity (FC). However, as we know, there were few studies to explore the impact of needling KI3 on brain large-scale FC in healthy people with different ages, which is essential for understanding the mechanism of needling.

The question of how needling at acupoint KI3 can produce different effects on different people in brain functional connectivity has been a matter of interest in the present study. Importantly, previous regional homogeneity (ReHo) analysis [11] found that after acupuncture at KI3 of healthy subjects, the ReHo value of Brodmann area (BA) 7 decreased. However, another similar ReHo analysis [12] showed that after real acupuncture at KI3, ReHo values were increased in the right sublobar region and BA 10 and were decreased in BA 31. Considering that these studies employed volunteers with diverse age ranges, we may attribute the result inconsistencies between different studies to the demographic data. It is, therefore, reasonable to expect that the brain FCs can be heterogeneous between healthy youths and elders after acupuncture at KI3.

Currently, numerous approaches have been developed and used to study the resting-state fMRI, such as the amplitude of low-frequency fluctuation (ALFF) [13], fractional ALFF (fALFF) [14], voxel-mirrored homotopic connectivity (VMHC) [15], ReHo [16], and large-scale FC. These clustering solutions may reveal physiological or pathological effects from different layers. However, some studies have recognized that ReHo analysis achieved better performance in depict clinical trait than ALFF or fALFF [17, 18]. In addition, VMHC focuses on exploring the differences in homotopic coordination (e.g., sex differences), not the whole-brain network. Moreover, large-scale FC are collections of widespread brain regions showing functional connectivity, which provide a coherent framework for understanding functional changes by offering a neural model of how different functions emerge when different conditions of intervention are adopted. Therefore, in the present study, we applied ReHo and large-scale FC analysis to test the hypothesis that acupuncture at KI3 in young and elderly people could induce heterogeneous acupuncture effects. This is practically considering that in the future, we may adopt different stimulation protocols when encountering the same conditions in youth and elder.

## 2. Material and Methods

### 2.1. Participants

The volunteers in the study were recruited in the First Affiliated Hospital of Southwest Medical University. Healthy elder volunteers were assessed by complete physical and neuropsychological examinations including Mini-Mental State Examination (MMSE) and Montreal Cognitive Assessment (MoCA). The inclusion criteria for the two groups are as follows: (1) right-handed, (2) regular diet and normal sleep patterns, (3) no neurological or psychiatric disorders reported, (4) no drug dependence and alcohol addiction, (5) moderate weight (BMI is 18.5–23.9), and (6) no brain lesions were observed by a routine magnetic resonance imaging (MRI) scan. The study was approved by the Medical Ethics Committee of the First Affiliated Hospital of Southwest Medical University (approval number: KY2019007).

### 2.2. Image Acquisition

**Figure 1:**
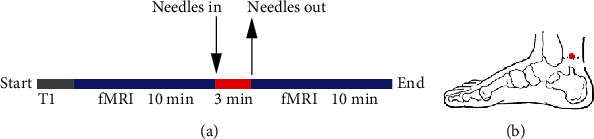
Trial flowchart (a) and location of acupoints KI3 (b).

The fMRI data acquisition was performed with a 3.0 Tesla (MRI Achieva, Philips Medical Systems, Nederland) MRI scanner using echo-planar imaging (EPI) sequence (TR 2000 ms, TE 30 ms, matrix 64∗64, FOV 240∗240∗152, voxel size 3.75 mm∗3.75 mm∗4 mm, flip angle 90°, 38 slices, orientation transverse, scan order interleaved, slice thickness 4 mm, gap 0, duration 546 s). Foam padding and earplugs served to control head motion and reduce the influence of scanner noise during the scanning. And subjects were told to hold still, keep their eyes closed, and think of nothing in particular. Before examination, the volunteers were instructed to rest for 20 minutes and were informed of the whole experimental procedure. The subjects first underwent a structural 3D T1-weighted scan (TR 8 ms, TE 4 ms, 256∗256 matrix, flip angle 7°, voxel size 1 mm∗1 mm∗1 mm, slices 160, slice thickness 2 mm) covering the whole brain. Then, fMRI data of resting state before needling were obtained (270 time points). After that, 3-minute-acupuncture stimuli on KI3 were performed. Subsequently, a second fMRI data (270 time points) in the postneedling state was acquired (Figure 1).

### 2.3. Acupuncture Intervention

All acupuncture operations were performed by the same acupuncturist with more than 5 years of clinical experience. The location of KI3 was set out in accordance with Name and Location of Acupoints: Chinese National Standards GB12346-90 (2006). Disposable sterile acupuncture needles (0.25 mm∗30 mm, Zhongyan, Beijing, China) were utilized for the acupuncture. The needles were inserted perpendicularly to a depth ca. 2 cm at KI3. To induce and enhance the needling (de qi) sensation, the acupuncturist manipulated the inserted needle by rotating the needle clockwise and counterclockwise with + or -360 degrees in each rotation, and lifting-thrusting of a needle. The acupuncturist performed each rotating and lifting-thrusting alternately with each lasting for 30 seconds. The two methods of needle manipulation were performed for 3 minutes in all.

### 2.4. Data Preprocessing

Initial resting-state fMRI statistics were preprocessed using Data Processing Assistant for Resting-State fMRI 2.3 (DPARSF. http://www.restfmri.net/forum/DPARSF) [19] which builds on Statistical Parametric Mapping (SPM8, http://www.fil.ion.ucl.ac.uk/spm). The preprocessing steps included in the study were as follows: we converted the original data of Digital Imaging and Communications in Medicine (DICOM) format to NIfTI format; then, the first 4 time points were discarded; the remaining 266 time points were corrected for slice timing to the middle slice and were realigned to correct for head movements; subjects with more than 2 mm displacement in any of the *x*, *y*, or *z* directions were excluded from this study. After that, the Friston 24-parameter model [20] was employed to regress head motion effects out of the realigned data; meanwhile, linear trends, white matter, cerebrospinal fluid (CSF) signal, and global average signal were regressed by multiple linear regression analysis; subsequently, the Diffeomorphic Anatomical Registration Through Exponentiated Lie Algebra (DARTEL) [21] tool was used to compute the transformations from native individual space to Montreal Neurological Institute (MNI) space with a voxel size of 3∗3∗3 mm; the voxel-specific framewise displacement (FD) of each subject was computed using Jenkinsons' method [22, 23]. And the mean Jenkinson FD of each subject was used as a covariate in the group comparisons of ReHo. Finally, we performed temporal bandpass filtering (0.01-0.08 Hz) across time series. After preprocessing, data from 20 subjects in resting state and postneedling state functional images were contained in statistical analyses. These preprocessed data without smooth were used in the later ReHo analysis.

Gaussian spatial smoothing was performed (FWHM = 4 mm) for the functional data in the later large-scale FC analysis.

### 2.5. ReHo Analysis

After data preprocessing, ReHo analysis, using the brain function of data processing software named DPARSF to calculate the whole brain around each individual element and its adjacent 27 individual elements on the time series of consistency, get the voxel KCC; the whole brain around each individual element KCC values divided by the whole brain of all voxel KCC get standardized ReHo diagram of mean values. Finally, the ReHo graph is smoothed, that is, an isotropic Gaussian kernel with FWHM of 6 mm is convolved to improve the signal-to-noise ratio [16, 24].

### 2.6. Large-Scale Functional Connectivity Analysis

After the fMRI data preprocessing, we used MATLAB toolbox GRETNA (http://www.nitrc.org/projects/gretna/) [25], a graphics theory network analysis toolbox of image connectivity, to build functional brain networks. The Power 264 atlas [26] produced by Power et al. and used for the primary analysis was employed as a template to extract the time series. Power 264 functional ROIs was composed of 14 functional networks including sensory/somatomotor hand, sensory/somatomotor mouth, cingulo-opercular task control, auditory, default mode, memory retrieval, ventral attention, visual, frontoparietal task control, salience, subcortical, cerebellar, dorsal attention, and uncertain. In this study, the whole cerebral cortex was divided into 264 cortical and subcortical regions by using this template. The mean time series of each node was extracted. Then, pairwise functional connectivity was estimated among the time series by calculating linear Pearson correlation coefficients. After that, the mean intra- and internetworks of FC were calculated by using the mean value of edges in corresponding subnetworks.

### 2.7. Functional Relevance Analysis of ReHo Results

To determine the functional relevance of ReHo difference regions, the Behavioral Analysis tool in the public domain Multi-image Analysis GUI (MANGO; Research Imaging Institute, UTHSCSA, San Antonio) software was employed (see method description [27]). The Behavioral Analysis performs regional behavior analysis based on user-defined ROIs by utilizing data from the BrainMap database (http://www.brainmap.org). In this study, we defined ROIs based on the clusters of ReHo results. The ROIs have been normalized to Talairach space and been loaded into MANGO. The Behavioral Analysis tool then automatically listed each behavioral subdomain associated with loaded ROI and calculates the associated *z*-score for each behavior. A positive *z*-score larger than 3.0 was considered significant after a Bonferroni correction for multiple comparisons (*P* < 0.05 with Bonferroni correction for multiple comparisons) [27].

### 2.8. Statistical Analysis

A paired *t* test was used to compare the ReHo results between postneedling and resting state in healthy youth and elder groups, respectively. And the mean Jenkinson FD was used as a covariate to eliminate the disturbance of microhead motion. The statistical maps were GRF corrected at a voxel level *P* < 0.001 and cluster level *P* < 0.05 (one-tailed). Edge connected cluster connectivity criterion, rmm = 5. Then, using a MATLAB toolbox xjview (http://www.alivelearn.net/xjview) with Anatomical Automatic Labeling template 2 (AAL2) [28] mapped the location of voxels that had significantly different ReHo values between conditions on MNI coordinate space.

A paired *t* test was used to compare changes of functional network connection between postacupuncture state at KI3 and resting state in the youth group and the elder group, respectively. False discovery rate (FDR) correction with a threshold *P* < 0.05 was used for multiple comparisons. Cohen's *d* value was calculated to depict the effect size between two variables.

## 3. Results

### 3.1. Sample Composition

Twenty healthy young (aged 24.7 ± 2.9, HY) and twenty healthy elderly (aged 56.8 ± 7.2, HE) right-handed volunteers were included in the study. The mean Jenkinson FDs of the youth and elder groups showed no significant differences in both resting state and postneedling state. The characteristics of the subjects in healthy youth and elder groups are shown in Table 1.

### 3.2. Different ReHo Alterations after Needling KI3 in HYs and HEs

**Figure 2:**
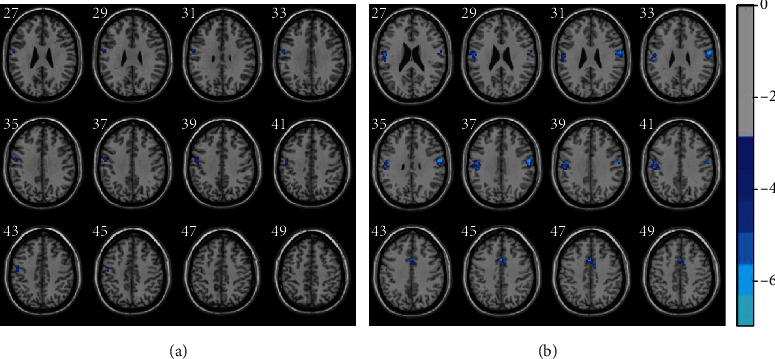
The outcome of ReHo differences after acupuncturing at KI3 in the healthy youth group (a) and the healthy elder group (b) compared with their own resting-state fMRI. The ReHo maps were shown in the MNI space. The ReHo maps were calculated using the paired *t* test and GRF correction at a voxel level *P* < 0.001 and cluster level *P* < 0.05 (one-tailed). Edge connected cluster connectivity criterion, rmm = 5. The cluster assignment has been shown in Table 2. The numbers in the figure indicated the *z*-axis in the MNI space. The color bar in the right panel indicated the intensity value of corresponding voxels.

The data of postneedling state fMRI in both the HY group and the HE group were compared to their own data of resting-state fMRI. Three clusters (5 structures) and 5 clusters (10 structures) were yielded in the HY group and the HE group, respectively. The most striking finding is that the largest altered ReHo difference regions between postneeding state and resting state are located in the occipital and frontal lobe in the HY group. In the HE group, however, the largest altered ReHo difference regions are located in the parietal and frontal lobe. More specifically, it was noted that some brain regions including left postcentral, right paracentral lobule, and right SMA in both groups showed decreased ReHo after acupuncture. Moreover, compared with resting state, brain regions involving right lingual and precentral present declined ReHo in the HY group. In addition, downgraded ReHo after needling KI3 in the HE group were found in right postcentral and precentral, left supramarginal gyrus and SMA, and both cingulum middle (Figure 2, Table 2).

### 3.3. HYs and HEs Showing Different Changes of Intra- and Internetwork FC Postneedling

**Figure 3:**
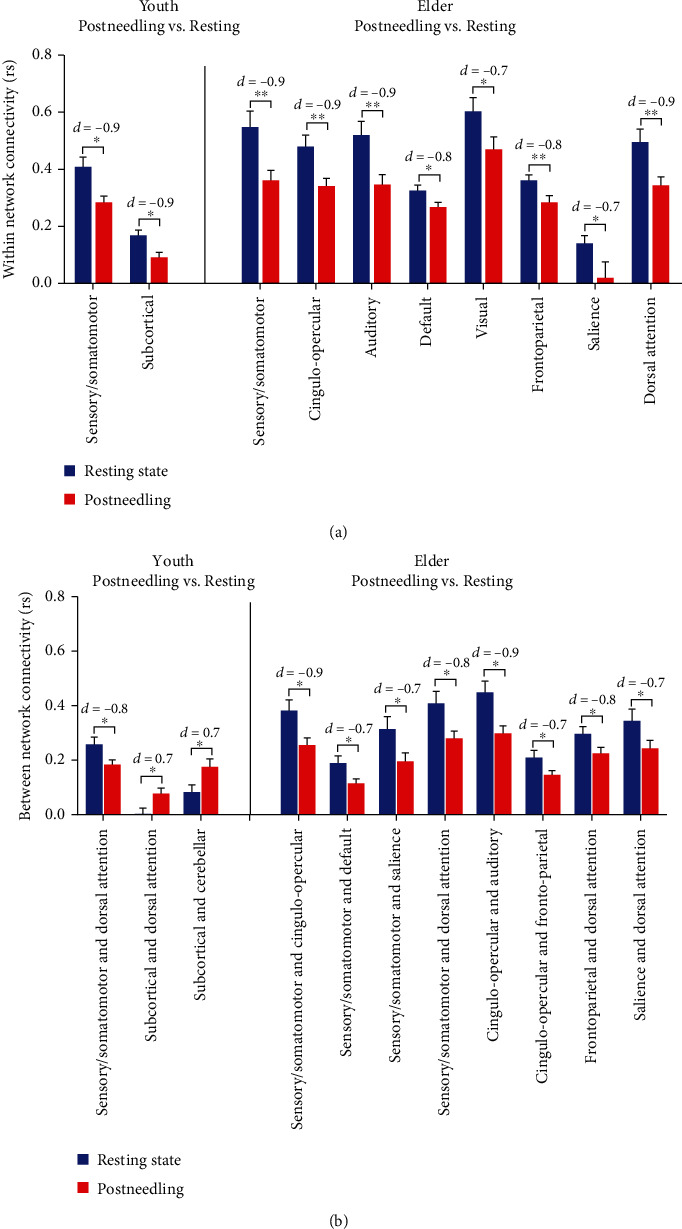
Average within and between subnetwork connections of fMRI in postneedling KI3 (red bar) compared with resting state (blue bar). (a) Average within network connections of subnetworks after needling KI3 compared to resting state in the youth group and the elder group. (b) Average between network connections of subnetworks after needling KI3 compared to resting state in the healthy youth group and the elder group. Those within or between subnetwork connections that showed no differences were not displayed in the figure. ^∗^*P* < 0.05 and ^∗∗^*P* < 0.01 after FDR correction. The *d* value in upper part of the bars indicated Cohen's *d* value, which depicted the effect size of connections between two groups.

After preprocessing of the neuroimaging data, time series for the Power 264 functional ROIs of each subject were extracted. Then, the average intra- and internetwork connections for each functional network of individuals were computed. And the intra- and internetwork FC in the postneedling state and resting state were compared in the HY group and the HE group, respectively (Figure 3).

### 3.4. HY Group

Compared with the resting state, the HY group in the postneedling state showed lower mean intranetwork FC in sensory/somatomotor (FDR *P* < 0.05, *d* = −0.9) and subcortical network (FDR *P* < 0.05, *d* = −0.9). In addition, the internetwork FC between sensory/somatomotor and dorsal attention had significantly decreased after acupuncture (FDR *P* < 0.05, *d* = −0.8). Furthermore, the internetwork FC between subcortical and dorsal attention (FDR *P* < 0.05, *d* = 0.7) and subcortical and cerebellar (FDR *P* < 0.05, *d* = 0.7) showed the most obvious elevations after needling in the HY group (Figure 3).

### 3.5. HE Group

Large-scale FC analysis indicated that compared with resting-state fMRI, both FCs of internetwork and intranetwork were declined after needling in the elder group. We found that the average connections of intranetwork such as sensory/somatomotor (FDR *P* < 0.01, *d* = −0.9), cingulo-opercular (FDR *P* < 0.01, *d* = −0.9), auditory (FDR *P* < 0.01, *d* = −0.9), default (FDR *P* < 0.05, *d* = −0.8), visual (FDR *P* < 0.05, *d* = −0.7), frontoparietal (FDR *P* < 0.01, *d* = −0.8), salience (FDR *P* < 0.05, *d* = −0.7), and dorsal attention (FDR *P* < 0.01, *d* = −0.9) networks significantly decreased in HEs after acupuncture.

The effect of acupuncture on internetwork FC primarily involved sensory/somatomotor, cingulo-opercular, and dorsal attention networks. It was noted that the internetwork FC between sensory/somatomotor and cingulo-opercular (FDR *P* < 0.05, *d* = −0.9), default (FDR *P* < 0.05, *d* = −0.7), salience (FDR *P* < 0.05, *d* = −0.7), dorsal attention (FDR *P* < 0.05, *d* = −0.8), between cingulo-opercular and auditory (FDR *P* < 0.05, *d* = −0.9), frontoparietal (FDR *P* < 0.05, *d* = −0.7), between frontoparietal and dorsal attention (FDR *P* < 0.05, *d* = −0.8), and between salience and dorsal attention (FDR *P* < 0.05, *d* = −0.7) were significantly declined after needling in the HE group (Figure 3).

### 3.6. Functional Relevance Differences of ReHo Clusters from HYs and HEs

To further explore the heterogeneous acupuncture effects of KI3 on HYs and HEs, we defined the ReHo clusters as ROIs and performed behavioral domain analysis. We found that the functions of ROIs from both HYs and HEs are involved in Action/Execution (speech), Action/Execution (unspecified), and Cognition/Language (speech). However, Cognition/Language (semantics) was only observed in clusters from the HY group. Perception/Somesthesis (pain) and Perception/Somesthesis (unspecified) were only obtained in clusters from the HE group (Table 3).

## 4. Discussion

In the present study, we applied ReHo and large-scale FC analysis to detect the acupuncture effects in HY and HE groups. We found that after needling, both HY and HE groups showed significant changes in ReHo and large-scale FC, compared with the resting state. Strikingly, stimulation at the same acupoint KI3 could cause some different acupuncture effects between youth and elder groups. Our study demonstrated that there are divergences in acupuncture effect when stimulating the same acupoint in subjects with different conditions, which would be practical in the stimulation strategies selecting in the future.

By ReHo analysis, we found that both HEs and HYs showed decreased ReHo in the postcentral gyrus, SMA, and PCL after needling KI3. This congruency in ReHo differential regions postneedling between HY and HE groups indicated that the method used in the present study was stable and reproducible. Meanwhile, considering that the postcentral gyrus played a prominent role in the sensory aspects of pain and a previous study has reported that the functions of KI3 involve pain relief [29, 30], moreover, SMA might receive higher regional cerebral blood flow across pain modalities [31], which supported that needling at KI3 had a synergistic effect on treating pain. We speculated that this result might be linked to the mechanism of needling KI3 in treating pain-related and cognition impairment diseases.

It was noted that HEs and HYs had different ReHo differential brain regions after needling KI3. Decreased ReHo in lingual and precentral in the HY group were found, while the ReHo after needling in the HE group decreased in postcentral, precentral, supramarginal gyrus, SMA, and both cingulum middle. Parts of these results were consistent with previous studies [12, 32, 33]. These findings further indicated that acupuncture of KI3 had different acupuncture effects in different age groups.

The different acupuncture effects of KI3 in HY and HE groups were reproduced in the latter large-scale FC analysis. There were more subnetworks with decreased connectivity after acupuncture in the average intranetwork of HEs. In addition, we found the average internetwork FC in the HY group including subcortical and dorsal attention, cerebellar increased, and sensory/somatomotor and dorsal attention decreased after needling acupoint. In the HE group, more average intra- and internetwork FC involving sensory/somatomotor, dorsal attention, cingulo-opercular, etc., were decreased by acupuncture. These results were consistent with previous fMRI studies, which suggested that acupuncture regulates the activity of some cortical and subcortical brain regions [34–36].

Notably, the enhanced average internetwork FC involving subcortical networks were not obtained in HEs and the HE group showed decreased internetwork FC mainly related to nonsubcortical networks such as sensory/somatomotor network, cingulo-opercular task control network, auditory network, and default mode network. These findings further supported acupuncture effects in different age groups. We might explain these results by the aging-related neuroplasticity. There were heterogeneous aging effects in the evolutions of FC with aging, and these changes in FC could be altered by external intervention [37–39].

From the functional relevance analysis, we found that the heterogeneous acupuncture effects in ReHo clusters might cause functional heterogeneity of acupuncture at KI3 between HY and HE groups. The functions of ROIs deprived from ReHo clusters of both groups were involved in Cognition and Action/Execution, while the function involving Perception/Somesthesis was only obtained in clusters from the HE group. These findings might indicate that acupuncture at KI3 could affect functions involving Cognition, Action/Execution, and Perception/Somesthesis. Given that changes of inhibition or excitation in brain regions played an important role in the functional evolution of aging [40–43], we may attribute the heterogeneous acupuncture effects between youth and elder groups to the changes of balance between inhibition and excitation. These findings further suggested the heterogeneous acupuncture effects of KI3 on different groups. More importantly, this study revealed the acupuncture effect of KI3 in different age groups from a neurological perspective, which will provide evidences for the application of acupuncture in prevention and treatment in the future.

One limitation in the study is that the subjects in this experiment are all healthy people. In the forthcoming studies, patients will be included as subjects to detect the acupuncture effects on brain functional network connectivity in the pathological state.

## 5. Conclusions

In this functional imaging approach based on ReHo and large-scale FC analysis for detecting the changes of ReHo and FC, we demonstrated that the acupuncture of KI3 had different acupuncture effects in different age groups. After needling KI3, significant differences in ReHo and FC are observed, which may suggest that the influence of acupuncture on the brain spontaneous activity and functional network are important mechanisms for the neurological effect of acupuncture. In conclusion, our study revealed the heterogeneous acupuncture effect of KI3 in different age groups from a neurological perspective, which will provide supporting evidence for the usage of acupuncture in prevention and treatment in the future.

## Figures and Tables

**Table 1:** Characteristics of the subjects in healthy youth and elder groups^∗^.

Characteristics	HY (*n* = 20)	HE (*n* = 20)	*P* value^∗^
Male : female	9 : 11	8 : 12	1.00
Age (years, mean ± SD)	24.7 ± 2.9	56.8 ± 7.2	<0.001
Resting FD	0.08 ± 0.04	0.09 ± 0.04	0.34
Postneedling FD	0.09 ± 0.04	0.12 ± 0.04	0.12
MMSE	\	27.4 ± 2.5	\
MoCA	\	25.1 ± 4.2	\

^∗^Chi-squared test was used in comparisons of sex ratio between the HY group and the HE group. Two sample *t* test was used in comparisons of age and FD. HY: healthy youth group; HE: healthy elder group; FD: Jenkinson framewise displacement; MMSE: Mini-Mental State Examination; MoCA: Montreal Cognitive Assessment.

**Table 2:** Clusters of ReHo differences between postacupuncture state and resting-state fMRI^∗^.

Group	Peak MNI coordinate			Cluster voxels	Peak intensity	Lobe	BA	Structure (voxels)
	*x*	*y*	*z*					
*Youth*								
Cluster 1	18	-90	-6	51	-6.90	Occipital	\	Lingual_R (37)
Cluster 2	-54	-6	36	38	-5.15	Frontal	6	Postcentral_L (28)
								Precentral_L (10)
Cluster 3	6	-27	63	38	-5.93	Frontal	6	PCL_R (18)
								SMA_R (15)
*Elder*								
Cluster 1	-48	-24	30	102	-4.94	Parietal	\	Postcentral_L (83)
							\	SupraMarginal_L (12)
Cluster 2	57	-9	33	64	-5.54	Frontal	\	Postcentral_R (61)
Cluster 3	-3	9	42	36	-5.12	Limbic	32	Cingulum_Mid_L (14)
								Cingulum_Mid_R (13)
Cluster 4	15	-39	69	62	-6.05	Parietal	3	Postcentral_R (38)
								PCL_R (15)
Cluster 5	15	-24	66	53	-5.81	Frontal	\	Precentral_R (10)
								SMA_R (26)
								SMA_L (9)

^∗^The location assignment has been done by using a MATLAB toolbox xjview (http://www.alivelearn.net/xjview). A paired *t* test was used to compare the ReHo results between postneedling and resting state in healthy youth and elder groups, respectively. The statistical maps were GRF corrected at a voxel level *P* < 0.001 and cluster level *P* < 0.05 (one-tailed). Edge connected cluster connectivity criterion, rmm = 5. Those structures with a size greater than or equal to 9 voxels were reported. PCL: paracentral lobule; SMA: supplementary motor area; SupraMarginal: supramarginal gyrus; Cingulum_Mid: cingulum middle.

**Table 3:** Positive associations of behavioral domain data from MANGO for regions of ReHo clusters^∗^.

Domain	Category	*z*-score
*Youth*		
Action	Execution (speech)	8.06
Cognition	Language (speech)	5.98
Action	Execution (unspecified)	5.25
Cognition	Language (semantics)	3.19
*Elder*		
Action	Execution (unspecified)	11.33
Action	Execution (speech)	9.65
Perception	Somesthesis (pain)	8.18
Perception	Somesthesis (unspecified)	7.62
Cognition	Language (speech)	3.51

## Data Availability

The data used to support the findings of this study are available from the corresponding author upon request.
